# Self-Assembled Molecular Complexes of 1,10-Phenanthroline and 2-Aminobenzimidazoles: Synthesis, Structure Investigations, and Cytotoxic Properties

**DOI:** 10.3390/molecules29030583

**Published:** 2024-01-24

**Authors:** Kameliya Anichina, Nikolay Kaloyanov, Diana Zasheva, Rusi Rusew, Rositsa Nikolova, Denitsa Yancheva, Ventsislav Bakov, Nikolai Georgiev

**Affiliations:** 1 Department of Organic Synthesis, University of Chemical Technology and Metallurgy, 8 Kliment Ohridski Blvd., 1756 Sofia, Bulgaria; kameliya_anichina@uctm.edu (K.A.); bakov@uctm.edu (V.B.); 2 Department of Organic Chemistry, University of Chemical Technology and Metallurgy, 8 Kliment Ohridski Blvd., 1756 Sofia, Bulgaria; nikolaykaloyanov@uctm.edu (N.K.); denitsa.pantaleeva@orgchm.bas.bg (D.Y.); 3 Laboratory of Reproductive OMICs Technologies, Acad. Kiril Bratanov Institute of Biology and Immunology of Reproduction, Bulgarian Academy of Sciences, 73A Tsarigradsko Shosse Blvd., 1113 Sofia, Bulgaria; zasheva.diana@yahoo.com; 4 Department of Structural Crystallography and Materials Science, Acad. Ivan Kostov Institute of Mineralogy and Crystallography, Bulgarian Academy of Sciences, Acad G. Bonchev Str., Build. 107, 1113 Sofia, Bulgaria; r.rusev93@gmail.com (R.R.); rosica.pn@clmc.bas.bg (R.N.); 5 Institute of Organic Chemistry with Centre of Phytochemistry, Bulgarian Academy of Sciences, Acad. G. Bonchev Str., Build. 9, 1113 Sofia, Bulgaria

**Keywords:** self-assembly, 1,10-phenanthroline, 2-aminobenzimidazoles, X-ray diffraction analysis, DFT, antitumor activity, in vitro DNA binding

## Abstract

Three new molecular complexes (phen)_3_(2-amino-Bz)_2_(H^+^)(BF_4_^−^)·3H_2_O **5**, (phen)_3_(2-amino-5(6)-methyl-Bz)_2_(H^+^)(BF_4_^−^)·H_2_O **6**, and (phen)(1-methyl-2-amino-Bz)(H^+^)(BF_4_^−^) **7**, were prepared by self-assembly of 1,10-phenanthroline (phen) and various substituted 2-aminobenzimidazoles. Confirmation of their structures was established through spectroscopic methods and elemental analysis. The X-ray diffraction analysis revealed that the crystal structure of **7** is stabilized by the formation of hydrogen bonds and short contacts. In addition, the molecular geometry and electron structure of molecules **5** and **6** were theoretically evaluated using density functional theory (DFT) methods. According to the DFT B3LYP/6-311+G* calculations, the protonated benzimidazole (Bz) units act as NH hydrogen bond donors, binding two phenanthrolines and a BF_4_^−^ ion. Non-protonated Bz unit form hydrogen bonds with the N-atoms of a third molecule phen. The molecular assembly is held together by π-π stacking between benzimidazole and phenanthroline rings, allowing for N-atoms to associate with water molecules. The complexes were tested in vitro for their tumor cell growth inhibitory effects on prostate (PC3), breast (MDA-MB-231 and MCF-7), and cervical (HeLa) cancer cell lines using MTT-dye reduction assay. The in vitro cytotoxicity analysis and spectrophotometric investigation in the presence of ct-DNA, showed that self-assembled molecules **5**–**7** are promising DNA-binding anticancer agents warranting further in-depth exploration.

## 1. Introduction

Cancer stands as one of the most perilous global diseases, accounting for an estimated 10.0 million deaths in 2022 [1]. The prevalence of female breast cancer has now surpassed that of lung cancer, witnessing an estimated 2.3 million new cases, constituting 11.7% of all cancer cases. This is followed by lung (11.4%), colorectal (10.0%), prostate (7.3%), and stomach (5.6%) cancers. Prostate cancer, with nearly 1.4 million new cases and 375,000 deaths worldwide, ranked as the second most frequent cancer and the fifth leading cause of cancer-related deaths among men in 2020 [2].

Typically, cancer treatment methods revolve around the surgical removal of cancerous growths, accompanied by radiotherapy and chemotherapy. However, these methods carry a wide array of side effects and exhibit reduced efficacy due to the development of cell-resistant phenotypes in tumor cells and metastatic formations [3]. Hence, the urgent need arises for the exploration and development of new chemotherapeutic agents that can combat cancer with high efficiency and minimal adverse effects.

Nitrogen-based heterocyclic compounds play a pivotal role in contemporary medicinal chemistry, with extensive research dedicated to designing and synthesizing novel drug molecules, particularly those exhibiting anti-cancer properties. Electron-rich nitrogen heterocycles possess the ability not only to readily accept or donate protons but also to establish various weak interactions like hydrogen bonding, dipole-dipole interactions, van der Waals forces, and π-stacking interactions. This versatility enables them to bind with a diverse range of enzymes and receptors in biological targets [4].

The molecular hybridization approach stands out as an effective strategy in the design of anti-cancer drugs, creating new drug molecules containing multiple pharmacophores, each with a distinct mode of action. This strategy not only potentially reduces undesired side effects but also offers a pathway to overcome drug resistance [5,6].

**Figure 1:**
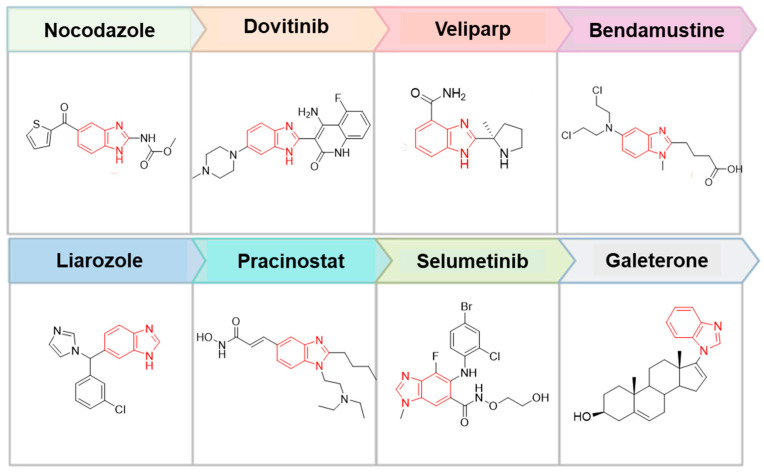
Chemical structures of some antineoplastic drugs containing benzimidazole moiety.

Among nitrogen-containing heterocycles, the benzimidazole scaffold, akin to the structural isostere of purine nucleotide bases, holds significance as a structural backbone in various clinically utilized antineoplastic drugs. Compounds like nocodazole, bendamustine, dovitinib, veliparip, pracinostat, liarozole, selumetinib, galeterone (Figure 1), among others, constitute part of this group [7,8,9,10]. Various benzimidazole derivatives with anticancer activity target different aspects of cancer cells, including DNA (through intercalation or alkylation), topoisomerases, poly (ADP ribose) polymerase (PARP), dihydrofolate reductase (DHFR), protein kinases and phosphatases, androgen receptors, and microtubules [7]. Consequently, the benzimidazole heterocyclic system emerges as an attractive platform for the development of new structures exhibiting antitumor activities.

**Figure 2:**
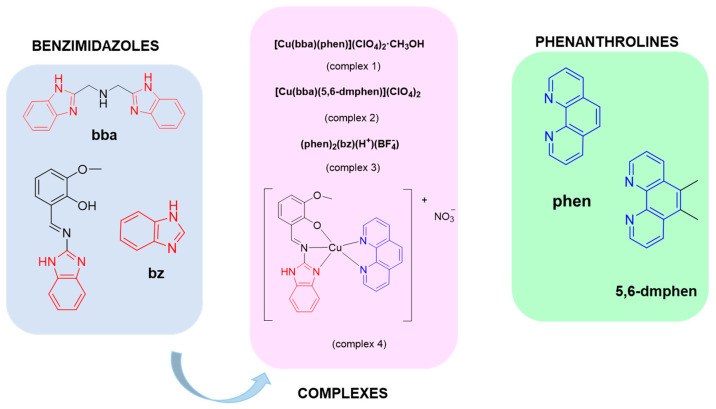
Anticancer complexes **1**–**4** incorporating benzimidazole and 1,10-phenanthroline moieties.

On the other hand, phenanthrolines are also important molecules in medicinal chemistry due to their intriguing biological and structural features [11,12]. Their metal complexes, together with supramolecular self-assembling structures, exhibit a range of biological activities including antimicrobial, antiviral, and antitumor effects [13,14,15,16,17,18]. Copper (II) complexes, specifically those with mixed ligands like 1,10-phenanthroline (phen) or 5,6-dimethyl-1,10-phenanthroline (5,6-dmphen) and benzimidazoles (complexes **1**, **2**, and **4**, Figure 2), emerged as promising candidates in the realm of anti-cancer drug discovery [19,20]. In our previous studies, we described in detail the synthesis of metal-free molecular complexes of phen and other nitrogen-containing heterocycles via self-assembly processes [21]. Compounds like [(phen)_2_(imidazole)(H^+^)(BF_4_^−^), (phen)_2_(benzimidazole)(H^+^)(BF_4_^−^) (complex **3**, Figure 2), and (phen)_3_(indole)(H^+^)(BF_4_^−^) exhibited significant cytotoxic effects in in vitro assays for antitumor activity against various human tumor cell lines, including HepG2 (liver carcinoma), HEp-2 (larynx epidermoid carcinoma), 8-MG-BA (brain tumor, glioblastoma multiforme), PC3 (prostate cancer), and MCF7 (breast cancer) [21,22].

Encouraged by these promising results, we present here the preparation of novel potential antitumor agents derived from the self-assembly of phen, 1*H*-benzimidazol-2-amine, and 2-aminobenzimidazoles substituted with a methyl group at the N-1 or C-5 (C-6) position of the benzimidazole (Bz) moiety and sodium tetrafluoroborate. Their structures were validated using FTIR, ^1^H NMR, elemental analysis, and single-crystal X-ray diffraction. Additionally, the molecular geometry and electron structure of these molecules were theoretically evaluated using density functional theory (DFT) methods. The molecular complexes were subjected to in vitro testing for inhibitory effects on tumor cell growth using MTT-dye reduction assay on specific cell lines including one prostate cancer cell line (PC3), two breast cancer human cell lines (MDA-MB-231 and MCF-7), one cervical cancer cell line (HeLa), and one normal skin foreskin cell line (BJ). The results of in vitro cytotoxicity indicate that the tested self-assembled benzimidazole-phenanthroline compounds are appropriate for further extensive exploration of their antitumor activity.

## 2. Results and Discussion

### 2.1. Chemistry

**Scheme 1:**
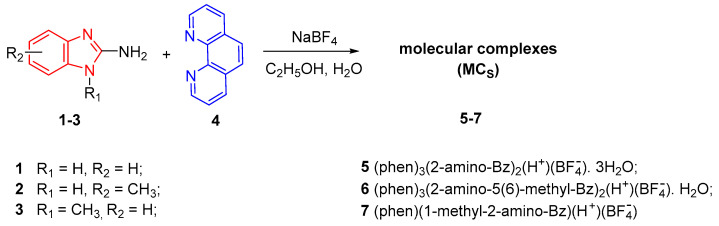
Synthesis of the studied self-assembled molecular complexes (MCs) **5**–**7**. Reagents and conditions: 96% C_2_H_5_OH/distilled water, 2–3 °C, 30 days.

The self-assembly to the target complexes **5**–**7** was carried out as shown in Scheme 1.

Following the effective synthetic approach [21], multicomponent structures were produced with the participation of 1,10-phenanthroline and different benzimidazole-2-amines such as 1*H*-benzimidazole-2-amine (Bz-2-amine), 5(6)-methyl-1*H*-benzimidazole-2-amine (5(6)-methyl-Bz-2-amine), and 1-methyl-benzimidazole-2-amine (1-methyl-Bz-2-amine. The complexes **5**–**7** are the result of a self-organizing process proceeding a month with the participation of corresponding 2-aminobenzimidazole, 1,10-phenanthroline, and NaBF_4_ in water-ethanol solution. As a result, we obtained molecular architectures in which the two units—phen and Bz are present in a 3:2 molar ratio in favor of phen when the benzimidazole-2-amine has a free N-H group in position 1 (compounds **5** and **6**). From an aqueous solution of phen, 1-methyl-Bz-2-amine, NaBF_4_, and ethanol we were able to isolate a complex with 1:1 stoichiometry (phen: 1-methyl-2-amino-bz). The three complexes contain counter ions—H^+^ and BF_4_^−^. The chemical structures of the new MCs were established by FTIR, ^1^H NMR spectra, and X-ray diffraction analysis as well as elemental analysis. The results are presented in the Section 3 and in the Supporting Material.

The FTIR spectra of **5**–**7** show the characteristic vibrational bands of each of the ligated species [21,23,24]. In the unassociated state of the benzimidazole ring, the IR bands for the NH stretching vibrations of the primary amino group are expected at ~3500 (symmetric) and ~3400 cm^−1^ (antisymmetric), while this of the secondary amino group—at ~3300 cm^−1^, respectively. Instead of three separate bands, the IR spectra of **5**–**7** show only one low-frequency shifted band, apparently due to strong hydrogen bonding. They were observed at 3396 (compound **5**), 3389 (compound **6**), and 3373 cm^−1^ (compound **7**). In the region 1690–1400 cm^−1^, there appeared strong bands for the C-N stretching vibrations and N-H deformation vibrations of the benzimidazole moieties. A very strong and broad band with several shoulders within the interval 1090–1030 cm^−1^ confirmed the presence of the BF_4_^−^ anions. Previously, for similar complexes [23], a characteristic IR band for the bending vibration of the 1,10-phenanthroline crystal water was observed at 1642 cm^−1^, but in the present case, the high intensity of the benzimidazole bands in this region hinders the detection of such a band.

In the ^1^H NMR spectra, measured in DMSO-*d*_6_, the signals of the phenanthroline moiety in MCs **5**–**7** were found around 9.10, 8.50, 8.00, and 7.78 ppm. The aromatic protons from the benzimidazole core resonated in the interval 7.50–6.88 ppm, while the methyl groups gave rise to singlets at 2.33 (complex **6**) and 3.61 ppm (complex **7**). The primary amino groups were identified by singlets shifting from 8.01 ppm to 7.48 ppm.

**Figure 3:**
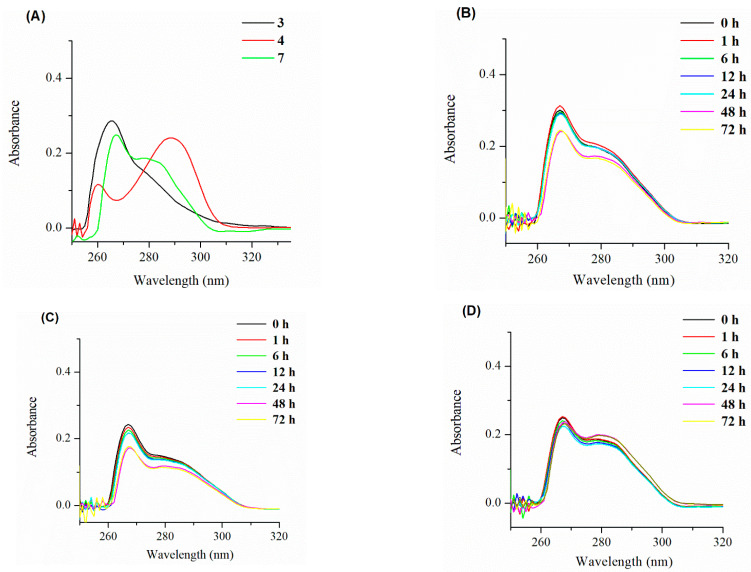
(**A**) Absorption spectra of (1-methyl-Bz-2-amine) **3**, 1,10-phenanthroline **4** and complex **7** in DMSO/water (9:1, *v*/*v* containing 10 mM Tris-HCl buffer pH 7.3) with concentrations of 1 × 10^−5^ mol/L. Changes in the absorption spectra with time of complexes **5** (**B**), **6** (**C**), and **7** (**D**) in DMSO/water (9:1, *v*/*v* containing 10 mM Tris-HCl buffer pH 7.3) with concentrations of 1 × 10^−5^ mol/L.

The stability of the synthesized complexes **5**–**7** at physiological pH was also considered a probable issue due to the possible destabilization and formation of the separate organic structures **1**–**4** from which the corresponding complexes were formed. In buffered solution DMSO/water (9:1, *v*/*v* containing 10 mM Tris-HCl buffer pH 7.3) the starting benzimidazoles **1**–**3** showed absorption spectra in a range between 250 nm and 310 nm with a maximum of 263 nm. In the same solution, 1,10-phenanthroline possessed an absorption band from 250 nm to 320 nm with well pronounced maximum at 289 nm. However, in **5**–**7** due to the complexation the absorption band of the initial benzimidazoles was slightly red shifted to 265 nm, while the 1,10-phenanthroline was blue shifted to 279 nm which was illustrated in Figure 3A where the absorption spectra of compounds **3**, **4**, and **7** were depicted as a typical example. The resulting difference in the complexes **5**–**7** absorption spectra allows a spectrophotometric investigation of the complexes’ stability in which the complex destabilization should result in the appearance of the former benzimidazole and phenanthroline bands at 263 nm and 289 nm, respectively. Therefore, the novel complexes were studied spectrophotometrically at physiological pH for three days. (Figure 3B–D) The observed results showed only minor changes in absorbance intensity and a lack of novel bands at 263 nm and 289 nm corresponding to the separated benzimidazole and 1,10-phenanthroline moieties. Hence it could be concluded that the studied complexes were stable under these conditions.

### 2.2. Single-Crystal X-ray Diffraction Analysis

**Figure 4:**
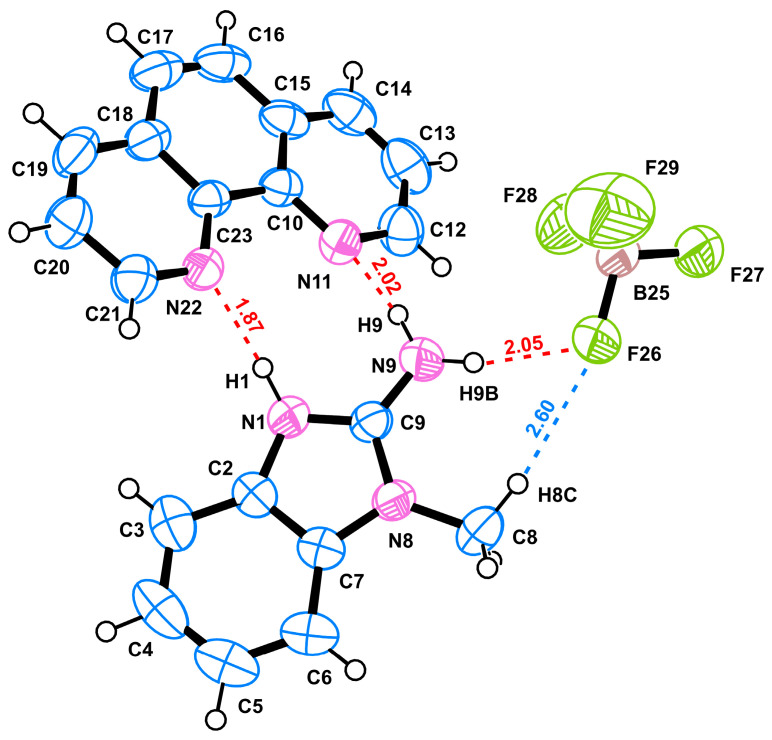
ORTEP view of the molecules present in the ASU of complex **7** (phen)(1-CH_3_-2-amino-Bz)(H^+^)(BF_4_^−^) with appropriate labeling scheme. The thermal ellipsoids are given with 50% probability level and the hydrogen atoms as spheres with arbitrary radii. The detected hydrogen bonding interactions and short contacts stabilizing the complex are visualized with red and blue dashes, respectively. The H-A distances are given in Å.

Colorless plate-shaped crystals of **7**, suitable for single crystal XRD analysis, were obtained directly from the EtOH/H_2_O reaction mixture. Complex **7** crystallizes in the monoclinic *P*2_1_/*n* space group with unit cell parameters *a* = 7.284 Å, *b* = 18.529 Å, *c* = 14.438 Å and β = 97.336° (Table S1). The molecules present in the asymmetric unit of the crystal structure of **7** (Figure 4) are 1-methyl-1*H*-benzimidazol-2-amine, 1,10-phenanthroline, and a tetrafluoroborate anion organized together as a self-assembled molecular complex. At a molecular level, both 1-methyl-1*H*-benzimidazol-2-amine and 1,10-phenanthroline moieties are planar with RMSD values of 0.022 Å and 0.025 Å, respectively. The orientation of the two moieties with respect to each other is expressed in plane normal to plane normal angle of 33.89° and plane to plane twist and fold angles of 33.24° and 7.03°, respectively.

**Figure 5:**
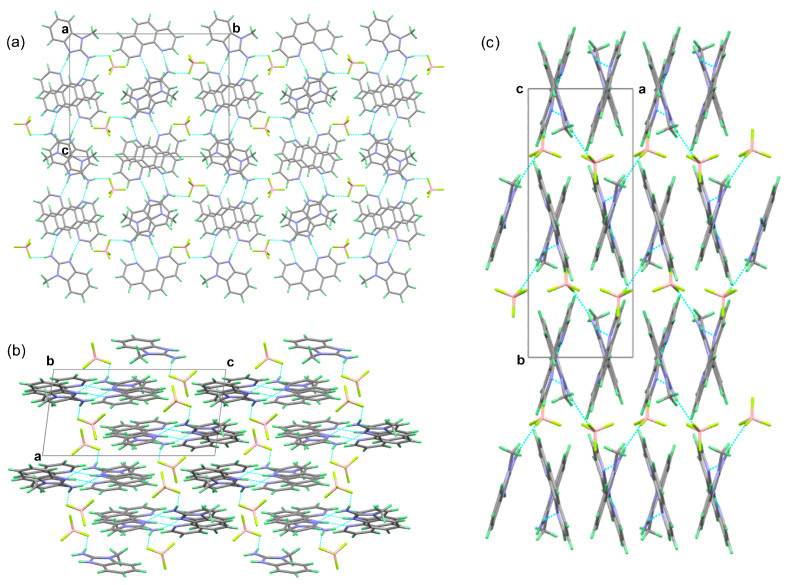
Three-dimensional packing of the molecules present in the crystal structure of **7**—(**a**) view along the *a* axis, (**b**) view along the *b* axis, and (**c**) view along the *c* axis. Hydrogen atoms are given in light green for clarity.

The molecular complex of **7** is stabilized predominantly by hydrogen bonding interactions of N-H…N, N-H…F type (Figure 4 and Table 1), but also by a plethora of short contacts (with distances < sum of vdW radii) and π-π stacking interactions. A total of three hydrogen bonding interactions are detected: two moderately strong between the 1-methyl-1*H*-benzimidazol-2-amine and 1,10-phenanthroline molecules (namely N1-H1…N22 and N9-H9A…N11) and another one between the fluorine atom F26 of the tetrafluoroborate and the 1,10-phenanthroline (namely N9-H9B…F26). Among the short contacts, the most pronounced is the C-H…F contact (considered a halogen interaction) between the F26 atom of the tetrafluoroborate and the H8C of the methyl group of 1,10-phenanthroline. Furthermore, the π-π stacking interactions are expected considering the presence of both the 1-methyl-1*H*-benzimidazol-2-amine and 1,10-phenanthroline conjugated systems. The combination of hydrogen bonding, short contacts, and π-π stacking interactions all contribute to the specific three-dimensional arrangement of the molecules in the crystal structure of complex **7** (Figure 5).

### 2.3. DTF Study

**Figure 6:**
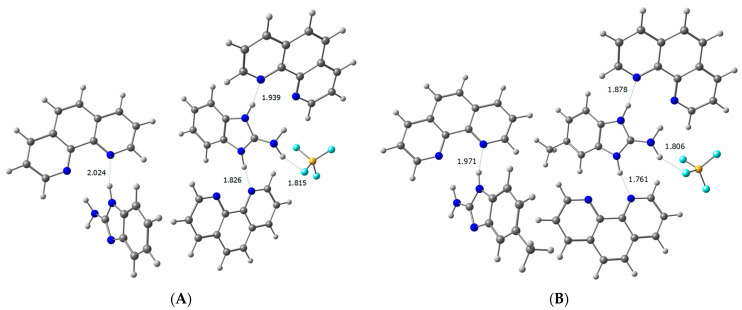
Gas-phase optimized geometry of complex **5** (**A**), and complex **6** (**B**), computed at B3LYP/6-311+G* level of theory. The following colors are used to denote the atoms: C—grey, N—blue, B—yellow, F—light blue. The bond distances are provided in Å.

The intermolecular interactions in complexes **5** and **6** were modeled by DFT B3LYP/6-311+G* calculations. The plausible geometry of the two complexes was constructed by considering the stoichiometry established for **5** and **6** by elemental analysis and NMR spectra on one side, and the supramolecular motifs found in the X-ray structure of complex **7**, on the other side. The optimization showed that a very similar pattern of intermolecular interactions can exist in both cases—Figure 6A,B.

The protonated benzimidazole moieties can act as NH hydrogen bond donors, binding two phenanthrolines and a BF_4_ ion. The nonprotonated benzimidazole unit can form two hydrogen bonds with the N-atoms of a third molecule phenanthroline. The two parts of the molecular assembly are held together by π-π stacking between the benzimidazole and phenanthroline rings. Arranged in this way, the molecules in the assembly have at least two N-atoms free to associate with water molecules.

According to the theoretical calculations, the introduction of a methyl group in the benzimidazole moiety (complex **6**, Figure 6B) results in shorter N-H…N distances and, therefore, stronger hydrogen bonding between the benzimidazole and the phenanthroline units, as well as with the BF_4_^−^ ion.

### 2.4. Cytotoxic Activity

The cytotoxicity effects of self-assembled MCs **5**–**7** on four human cell lines of reproductive tissue origin—MCF-7 (estrogen-responsive) and MDA-MB-231 (triple-negative, i.e., negative for estrogen receptor (ER), progesterone receptor (PR) and human epidermal growth factor receptor 2 (HER2)) breast cancer cell lines (both adenocarcinomas), PC3 (prostate), and HeLa (cervical) cell line as well as on a normal cell line BJ (skin fibroblasts), were studied employing a standard MTT assay. The complexes were evaluated in the concentration range of 0.4—400 μg/mL.

**Figure 7:**
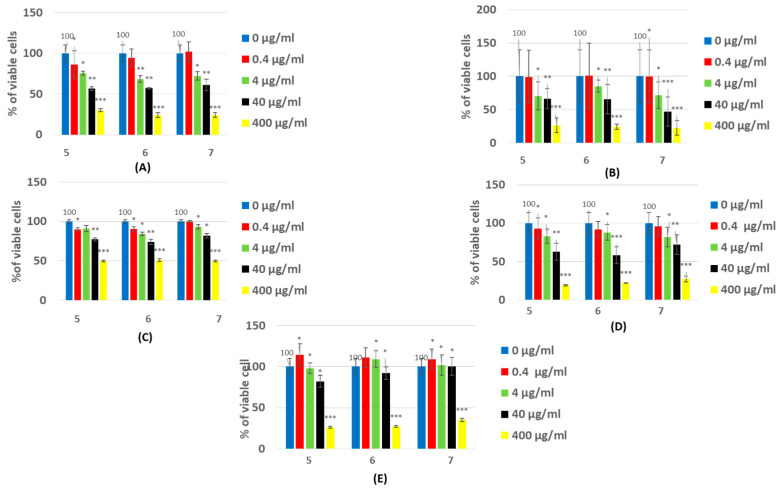
Cell viability assay conducted on tumoral MCF-7 (**A**), MDA-MB 231 (**B**), PC3 (**C**), HeLa (**D**) cell lines, and BJ (**E**) normal cell line treated with molecular complexes **5**–**7** after 24 h exposure as assessed by MTT-dye reduction assay. All groups are compared to the control. Statistical analysis utilized an ANOVA one-way test and a Tukey posttest (* *p* < 0.05; ** *p* < 0.01; *** *p* < 0.001).

Cytotoxicity towards the aforementioned cell lines corresponding to cell survival after 24 h of incubation with the studied compounds **5**–**7** was presented as a percentage of the cell viability of the positive control—non-treated cells (Figure 7).

The self-assembled compounds **5**–**7** exhibited comparable cytotoxicity on the tested tumor cell line MCF-7. For instance, when administered at a concentration of 40 µg/mL, MCs **5** and **6** led to 57% cell viability in MCF-7 cells after 24 h of incubation, while complex **7** resulted in 61% viability (Figure 7A). The highly invasive cell line MDA-MB 231 displayed reduced sensitivity to benzimidazole-phenanthroline derivatives **5**–**7** in comparison to MCF-7 cells, showcasing viability rates between 74–82% after 24 h of treatment with 40 µg/mL of MCs (Figure 6B). Notably, even at a concentration as high as 400 μg/mL, the tested complexes didn’t decrease cell survival below 50% in this particular tumor line (Figure 7B). These cytotoxic effects might be linked to the aggressive tumor phenotype(s) characteristic of the triple-negative breast cancer MDA-MB-231 (ER, PR, and HER2 negative) cell line [25]. Furthermore, MDA-MB-231 cells differ from other breast cancer cells due to the unique presence of several proteins, including MKP3 and Nedd4 [26]. Additionally, these cells have higher phosphorylated ERK1/2 levels compared to other breast cancer cell lines [26] and exhibit very low basal Akt activity [27].

The prostate cell line used in the in vitro screening is androgen receptor-negative and negative for the tumor suppressor gene p53, rendering it particularly challenging to treat using conventional therapeutics [28]. At 40 μg/mL after 24 h, the PC3 cell viability was at the lowest (47%) when the PC3 cells were treated with compound **7** (Figure 7C).

In examining the cytotoxic effects of **5**–**7** on HeLa cervical cancer cells, the results after 24 h (Figure 7D) indicated that compounds **5** and **6** exhibited cell viability of around 60%, while **7** displayed a higher viability of 72%.

A beneficial observation was the minimal impact of the studied self-assembled molecules on the cell viability of BJ cells after a 24-h treatment ranging from 0.4 to 40 μg/mL (Figure 7E).

The IC_50_ values, defined as the half maximal inhibitory concentration, are presented in Table 2 for MCF-7, PC3, and HeLa cell lines. The tested MCs demonstrated cytotoxicity activity, with variable IC_50_ values ranging from 0.061 to 0.228 µM/mL in screened in vitro models after 24 h incubation (Table 2). The molecular architectures **5** and **6**, in which the phen and Bz units are present in a 3:2 molar ratio in favor of phen, exhibited higher cytotoxicity relative to MCF-7 and HeLa tumor cells, falling within the range of 0.061–0.085 µM/mL. This is in contrast to complex **7**, in which the phen and Bz units are present in a 1:1 molar ratio (0.150–0.228 µM/mL, Table 2), displaying lower cytotoxicity. However, complex **7** demonstrated the best cytotoxicity when tested against prostate tumor cells (0.082 µM/mL).

It is a well-known fact that structures featuring planar polyaromatic or heteroaromatic systems (including phenanthroline and/or benzimidazole-based compounds) interact reversibly with the DNA double helix [30,31,32,33]. This interaction occurs through the insertion of the planar chromophore between adjacent base pairs at the intercalation site, resulting in topological alterations in the double helix, such as unwinding and lengthening. The distortion of DNA spatial conformation disrupts numerous DNA-protein interactions and, as a consequence, has the potential to induce cell death [34].

**Figure 8:**
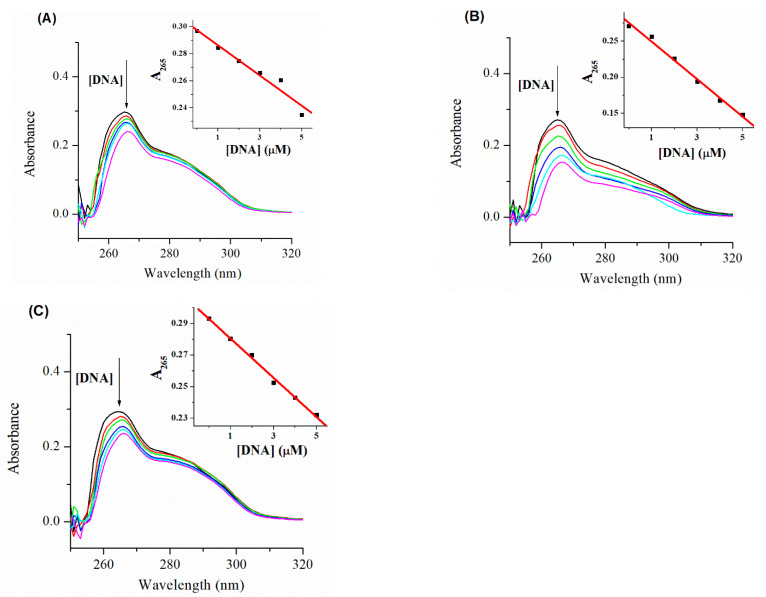
Changes in the absorption spectra of complexes **5** (**A**), **6** (**B**), and **7** (**C**) in DMSO/water (9:1, *v*/*v* containing 10 mM Tris-HCl buffer pH 7.3) with concentrations of 1 × 10 ^−5^ mol/L upon addition of ct-DNA. 0 μM DNA (black), 1 μM DNA (red), 2 μM DNA (yellow), 3 μM DNA (blue), 4 μM DNA (light blue) and 5 μM DNA (cyan).

Because self-assembled molecular complexes **5**–**7** are planar structures, it is possible that they interacted with DNA molecules. In order to confirm our statement about the possible interaction of the novel complexes with DNA molecules, a spectrophotometric investigation of **5**–**7** in the presence of calf thymus DNA (ct-DNA, Sigma-Aldrich, Steinheim, Germany) was performed in buffered solution DMSO/water (9:1, *v*/*v* containing 10 mM Tris-HCl buffer pH 7.3). It is well known that the bonding of DNA resulted in a decrease in absorption and fluorescence spectra of the organic compounds, which was usefully applied for the development of chemosensing molecules in the detection of DNA [32,33,35,36]. Similarly, complexes **5**–**7** showed a gradual decrease of its absorption spectra with the increased concentration of DNA (Figure 8). Moreover, the observed relation was linear (Figure 8 inset), which suggests a bounding process of the examined complexes and DNA molecules.

## 3. Materials and Methods

### 3.1. General Procedures

The reagents 1*H*-benzimidazole-2-amine, 97%; 1,10-phenanthroline monohydrate, 99%; and sodium tetrafluoroborate, 98% were obtained from Sigma-Aldrich (Steinheim, Germany). The other aminobenzimidazoles (5(6)-methyl-1*H*-benzimidazole-2-amine and 1-methyl-benzimidazole-2-amine) were synthesized by us according to procedure [37]. All commercially available solvents were obtained from Alfa Aesar (Heysham, UK) and were used without purification or drying. The melting points (mp) were measured using an Electrothermal AZ 9000 3MK4 apparatus (Stone, Staffordshire, UK) and were uncorrected. The composition of the products was determined by elemental analysis on Vario ELV5.18.018 (Elementar Analysensysteme GmbH, Hanau, Germany) performed in CHNS mode. IR spectra were recorded on a Bruker Tensor 27 FT spectrometer (Billerica, MA, USA) as potassium bromide discs. The ^1^H NMR spectra were recorded on a Bruker Avance 600 MHz spectrometer at room temperature (303 K) using DMSO-*d*_6_ as a solvent. Chemical shifts (δ) are reported in parts per million (ppm). Coupling constants J are given in Hertz (Hz), and spin multiplicities are given as singlet (s), broad singlet (br s), doublet (d), doublet of doublets (dd), triplet (t), triplet of doublets (td), and multiplet (m).

### 3.2. Chemistry

#### 3.2.1. General Procedure for Molecular Complexes **5**–**7** [21]

A solution containing 1,10-Phenanthroline monohydrate (0.435 mmoL), a corresponding 2-aminobenzimidazole derivative (0.435 mmol), 137 mg NaBF4 (2.5 mmoL), 3 mL of 96% EtOH and 20 mL distilled water was prepared at 70 °C. Subsequently, the solution was cooled to ambient temperature, and distilled H_2_O was added to reach a total volume of 25 mL. The resulting solution was then refrigerated at 2–3 °C for several days. The formed crystals were filtered, washed with distilled water, and finally dried in a desiccator over P_4_O_10._

#### 3.2.2. Compound Data

*(phen)_3_(2-amino-Bz)_2_(H^+^)(BF_4_^−^)·3H_2_O* (**5**). White powder (39%); mp 127–130 °C; FTIR (KBr, cm^−1^): 3396, 1682, 1640, 1512, 1270, 1084, 730. Anal. calcd. for C_50_H_45_N_12_O_3_BF_4_ (C, H, N): Calculated (%): C, 63.30; H, 4.78; N, 17.72. Found (%): C, 62.81; H, 4.34; N, 17.41. ^1^H-NMR (600 MHz, DMSO-*d*_6_), δ (ppm): 9.10–9.09 (dd, *J* = 1.80 Hz, *J* = 4.3 Hz, 6H, Phen-H); 8.50-8.49 (dd, *J* = 1.78 Hz, *J* = 8.00 Hz, 6H, Phen-H); 8.00 (s, 6H, Phen-H); 7.78-7.76 (dd, *J* = 4.27 Hz, *J* = 8.03 Hz, 6H, Phen-H); 7.55 (s, 4H, NH_2_); 7.26–7.23 (m, 4H, Ar-bz-H); 7.08-7.05 (m, 4H, Ar-bz-H).

*(phen)_3_(2-amino-5(6)-methyl-Bz)_2_H^+^BF_4_^−^·H_2_O* (**6**). White powder (Yield: 38%); mp 93–95 °C. FTIR (KBr, cm^−1^): 3389, 2924, 1683, 1643, 1509, 1277, 1083, 731. Anal. calcd. for C_52_H_45_N_12_OBF_4_ (C, H, N): Calculated (%): C, 66.39; H, 4.82; N, 17.87; Found (%): C, 65.89; H, 4.81; N, 17.57. ^1^H-NMR (600 MHz, DMSO-*d*_6_), δ (ppm): 9.10–9.09 (dd, *J* = 1.82 Hz, *J* = 4.28 Hz, 6H, Phen-H); 8.50–8.49 (dd, *J* = 1.80 Hz, *J* = 8.00 Hz, 6H, Phen-H); 8.00 (s, 6H, Phen-H); 7.78–7.76 (dd, *J* = 4.25 Hz, *J* = 8.20 Hz, 6H, Phen-H); 7.48 (s, 4H, NH_2_); 7.12–7.11 (d, *J* = 8.16 Hz, 2H, Ar-bz-H); 7.05–7.04 (m, 2H, Ar-bz-H); 6.89–6.88 (d, *J* = 7.95 Hz, 2H, Ar-bz-H); 2.33 (s, 6H, CH_3_).

*(phen)(1-methyl-2-amino-Bz)(H^+^)(BF_4_^−^)* (**7**). Colorless crystals (40%), mp 150–151 °C. FTIR (KBr, cm^−1^): 3373, 2903, 1685, 1515, 1233, 1073, 743. Anal. calcd. for C_20_H_18_N_5_BF_4_ (C, H, N): Calculated (%): C, 57.86; H, 4.37; N, 16.87. Found (%): C, 57.41; H, 4.27; N, 16.59. ^1^H-NMR (600 MHz, DMSO-*d*_6_), δ (ppm): 1H-NMR (600 MHz, DMSO-*d*_6_), δ (ppm): 9.11–9.10 (d, *J* = 3.06 Hz, 2H, Phen-H); 8.55–8.49 (m, 4H, Phen-H); 8.01 (s, 2H, NH_2_); 7.80–7.78 (dd, *J* = 4.45, *J* = 8.32 Hz, 2H, Phen-H; 7.51–7.49 (m, 1H, Ar-bz-H); 7.38–7.36 (m, 1H, Ar-bz-H); 7.29–7.25 (m, 1H, Ar-bz-H); 3.61 (s, 3H, CH_3_).

### 3.3. X-ray Structural Analysis

Colorless, plate-type single crystals of **7** (size~0.3 × 0.2 × 0.15 mm^3^) were mounted on a nylon loop using cryo-protective oil (Paraton N). Diffraction data were collected on a SupernovaDual diffractometer equipped with an Atlas CCD detector and a microfocus sealed Mo X-ray source (MoKα = 0.71073 Å). CrysAlisPro-ver.171.42.58a [38] was used as data collection and data processing software. The structure of 7 was solved with intrinsic phasing methods (ShelxT [39]) and refined by the full-matrix least-squares method on F2 (ShelxL [40]) both integrated in the OLEX2-ver.1.5 graphical interface [41]. All non-hydrogen atoms were located successfully from the Fourier map and were refined anisotropically. Hydrogen atoms were placed on calculated positions riding on the parent carbon atoms using the following scheme: Ueq = 1.2 for C-Haromatic = 0.93 Å and C-Hmethyl = 0.96 Å. The positions of the hydrogen atoms riding on a heteroatom (e.g., N-H and NH2) were refined from electron density maps. ORTEP-3v2 software [42] was used to depict the molecules present in the asymmetric unit. Mercury ver.4.0 (CCDC, [43]) was used to visualize the three-dimensional packing of the molecules in the crystal structure. The most important crystallographic data extracted from the single-crystal XRD experiment is given in Table S1. Bond lengths and bond angles for the crystal structure of **7** are given in Tables S2 and S3. Complete crystallographic data for the structure of **7** reported in this paper have been deposited in the CIF format with the Cambridge Crystallographic Data Center as 2311093. These data can be obtained free of charge via www.ccdc.cam.ac.uk/structures (accessed on 29 November 2023).

### 3.4. Computational Methods

Optimization of the structure of molecular complexes **5** and **6** was carried out by density functional theory (DFT) methods with the use of B3LYP functional [44] and 6-311+G* basis set implemented in the Gaussian 09 package [45]. The initial geometry for the optimization of the two complexes was constructed based on the established stoichiometry and intermolecular interactions found by the single-crystal X-ray study of complex **7**. The optimization was carried out in the gas phase without any symmetry constraints. Vibrational frequencies analysis was conducted to confirm the optimized geometries as true minima on the potential energy surface without imaginary frequency.

### 3.5. Cytotoxicity

#### 3.5.1. Cell Culture Methods

The human cell lines PC3 (ATCC^®^ CRL-1435^TM^), MCF-7 (ATCC^®^ N NTB-22^TM^), HeLa (ATCC^®^ CCL-2^TM^), and BJ (ATCC^®^ CRL-2522^TM^) were purchased from American Type Culture Collection (ATCC) (Manassas, VA, USA). PC3 and MCF-7 cells were cultivated in Dulbecco’s Modified Eagle Medium (DMEM, PAA Cell Culture Company, Cambridge, UK), supplemented with 10% fetal bovine serum (FBS, PAA) and antibiotic/antimycotic solution (PAA). BJ and HeLa cells were cultivated in Eagle’s Minimum Essential Medium (EMEM, PAA) supplemented with 10% FBS, 4 mM L-glutamine (Sigma-Aldrich, Steinheim, Germany) and antibiotic/antimycotic solution (PAA). The MDA-MB231 cell line was cultivated in a DMEM high glucose medium (Kibbutz Beit Haemek) supplemented with an amino acid solution (Sigma-Aldrich) and antibiotic/antimycotic solution (PAA).

#### 3.5.2. Cell Viability Assay

The cell viability was established using the MTT (3-(4,5-dimethylthiazol-2-yl)) method. This method is based on the reduction of MTT to purple formazan in the mitochondria of living cells. Its absorbance is measured by the Elisa Reader model FLUOstar OPTIMA (BMG Labtech) at 540 nm [46]. All cell lines were grown to the confluence of 70–80% and were trypsinized. They were counted in a Burker camera and 104 cells in a well were seeded in a 96-well plate. They were treated after 24 h with the complexes N1-N3 in 3–4 wells for each experiment and were incubated for 24 or 48 h. The untreated cells were used as a control with 100% viability. The results are scored on this base. All experiments are repeated in triplicate.

#### 3.5.3. Statistical Analysis

The one-way ANOVA test for each of the cell lines was treated with four different concentrations for 24 h and control was performed [47]. All results are statistically significant at *p* ≤ 0.05.

## 4. Conclusions

Three novel molecular complexes, **5**, **6**, and **7**, were prepared through the self-assembly of 1,10-phenanthroline and different substituted 2-aminobenzimidazoles. Structural confirmation was achieved via spectroscopic techniques and elemental analysis.

X-ray diffraction analysis unveiled that the molecular structure of complex **7** is stabilized by two moderately strong ones between the 1-methyl-1*H*-benzimidazol-2-amine and 1,10-phenanthroline molecules (namely N1-H1…N22 and N9-H9A…N11), and another involving the fluorine atom F26 of the tetrafluoroborate and the 1,10-phenanthroline (specifically N9-H9B…F26). Additionally, prominent short contacts were observed, notably the C-H…F contact (considered a halogen interaction) between the F26 atom of the tetrafluoroborate and the H8C of the methyl group of 1,10-phenanthroline. Moreover, the existence of π-π stacking interactions is expected due to the presence of both the 1-methyl-1*H*-benzimidazol-2-amine and 1,10-phenanthroline conjugated systems. The collective interplay of hydrogen bonding, short contacts, and π-π stacking contributes to the distinct three-dimensional arrangement of the molecules in the crystal structure of complex **7**.

Furthermore, the molecular geometry and electron structure of molecules **5** and **6** were theoretically examined using DFT methods. According to the DFT B3LYP/6-311+G* calculations, the protonated benzimidazole units act as NH hydrogen bond donors, binding two phenanthrolines and a BF4-ion. Non-protonated benzimidazole units form hydrogen bonds with the N-atoms of a third phen molecule. The molecular assembly is upheld by π-π stacking between benzimidazole and phenanthroline rings, allowing N-atoms to interact with water molecules.

Self-assembled complexes **5**–**7** underwent in vitro testing for their inhibitory effects on tumor cell growth in prostate (PC3), breast (MDA-MB-231 and MCF-7), and cervical (HeLa) cancer cell lines using the MTT-dye reduction assay. Notably, benzimidazole-phenanthrolines **5** and **6** revealed significant cytotoxicity against MCF-7 and HeLa tumor cells with IC_50_ values ranging from 0.061 to 0.085 µM/mL. However, complex **7** demonstrated the best cytotoxicity when tested against prostate tumor cells (IC_50_ = 0.082 µM/mL). From the spectrophotometric investigation conducted in the presence of calf thymus DNA in a buffered solution of DMSO/water, it can be concluded MCs **5**–**7** probably bind to the DNA molecules. These results position self-assembled structures **5** and **7** as promising DNA binding agents with antitumor activity, warranting further in-depth explorations.

## Figures and Tables

**Table 1:** Hydrogen bonding interactions and short contacts for complex **7**.

D	H	A	d(D–H), Å	d(H-A), Å	d(D-A), Å	D-H-A, °
C8	H8C	F26 ^1^	0.96	2.60	3.549(4)	169.6
N1	H1	N22	0.92(3)	1.87(4)	2.783(3)	170(3)
N9	H9A	N11	0.89(4)	2.02(4)	2.881(4)	162(3)
N9	H9B	F26 ^1^	0.87(4)	2.05(4)	2.895(4)	165(3)

Symmetry operation: ^1^ 1/2 + X, 1/2 − Y, 1/2 + Z.

**Table 2:** In vitro cytotoxicity of the benzimidazole-phenanthroline molecular complexes **5**–**7** against tumor MCF-7, PC3, and HeLa cell lines after 24 h incubation.

IC_50_ (µM/mL) ± SE ^a^			
Compound	MCF-7 Cells	PC3 Cells	HeLa Cells
**5**	0.085 ± 1.20	0.092 ± 1.57	0.064 ± 2.00
**6**	0.061 ± 1.42	0.148 ± 1.53	0.081 ± 1.38
**7**	0.150 ± 1.41	0.082 ± 2.00	0.228 ± 1.30
**Doxorubicin**	1.2 ± 0.005 [29]	N.D.	N.D.

^a^ SE—Standard error; N.D.—Not determined.

## Data Availability

Data are available within the article.

## Supplementary Materials

The following supporting information can be downloaded at: https://www.mdpi.com/article/10.3390/molecules29030583/s1, Figures S1a–S4. IR and NMR spectra of complexes; Table S1. The most important crystallographic parameters from the crystal structure refinement of complex **7**; Table S2. Bond lengths for the crystal structure of **7**; Table S3. Bond Angles for the crystal structure of **7**.
