# Supplementary material for: Self-Assembled Molecular Complexes of 1,10-Phenanthroline and 2-Aminobenzimidazoles: Synthesis, Structure Investigations, and Cytotoxic Properties
Source: Molecules. 2024 Jan 24;29(3):583. doi: 10.3390/molecules29030583 (PMC10856035; doi:10.3390/molecules29030583)
Supplement: Supplementary file 1 [file molecules-29-00583-s001.zip › molecules-2776621-supplementary.pdf]

Self-Assembled Molecular Complexes of 1,10-Phenanthroline and 2-Aminobenzimidazoles: Synthesis, Structure Investigations, and Cytotoxic Properties

Kameliya Anichina [1], Nikolay Kaloyanov[2], Diana Zasheva[3], Rusi Rusew[4], Rositsa Nikolova[4], Denitsa Yancheva [2,5], Ventsislav Bakov[1], Nikolai Georgiev[1]*

[1] Department of Organic Synthesis, University of Chemical Technology and Metallurgy, 8 Kliment Ohridski Blvd., 1756 Sofia, Bulgaria; kameliya_anichina@uctm.edu (K.A.); bakov@uctm.edu (V.B.)

[2] Department of Organic Chemistry, University of Chemical Technology and Metallurgy, 8 Kliment Ohridski Blvd., 1756 Sofia, Bulgaria; nikolaykaloyanov@uctm.edu (N.K.)

[3] Laboratory of Reproductive OMICs Technologies, Acad. Kiril Bratanov Institute of Bi-ology and Immunology of Reproduction, Bulgarian Academy of Sciences, 73A Tsarigradsko Shosse Blvd., 1113 Sofia, Bulgaria; zasheva.diana@yahoo.com (D.Z.)

[4] Department of Structural Crystallography and Materials Science, Acad. Ivan Kostov Institute of Mineralogy and Crystallography, Bulgarian Academy of Sciences, Acad G. Bonchev Str., Build. 107, 1113 Sofia, Bulgaria; r.rusev93@gmail.com (R.R.); rosica.pn@clmc.bas.bg (R.N.)

[5] Institute of Organic Chemistry with Centre of Phytochemistry, Bulgarian Academy of Sciences, Acad. G. Bonchev str. Bl. 9, 1113 Sofia, Bulgaria; denitsa.pantaleeva@orgchm.bas.bg (D.Y.)

* nikigeorgiev@uctm.edu (N.G.);

Contents

Figures. S1a-S4. IR and NMR spectra of complexes pages 2-5

Table S1. The most important crystallographic parameters from the crystal structure refinement of complex 7. page 6

Table S2. Bond lengths for the crystal structure of **7** page 7

Table S3. Bond Angles for the crystal structure of **7** page **8**

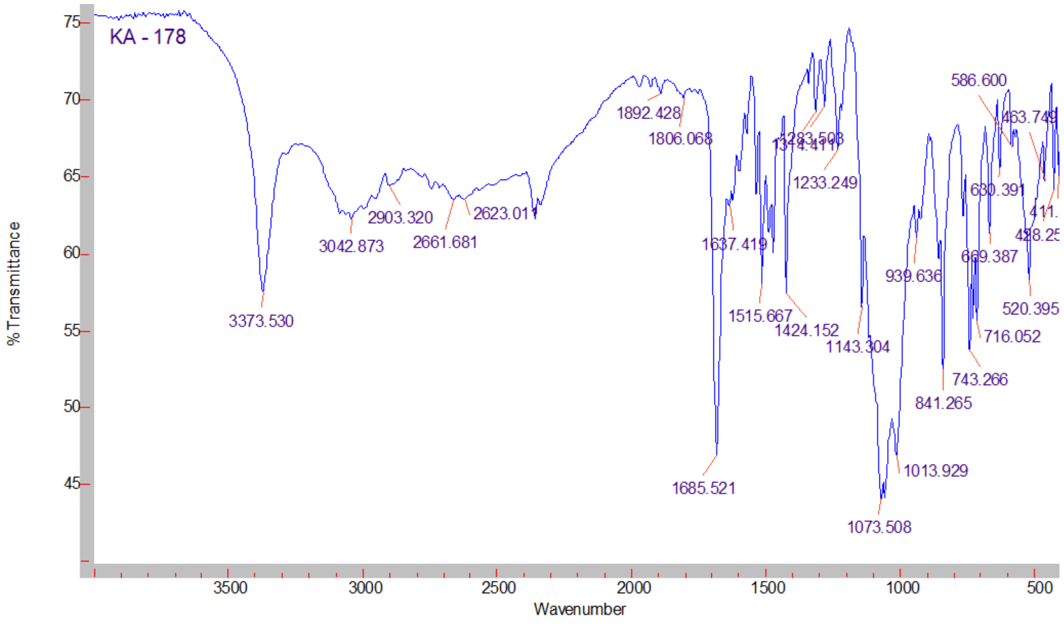

FigureS1a. IR spectrum of complex 7

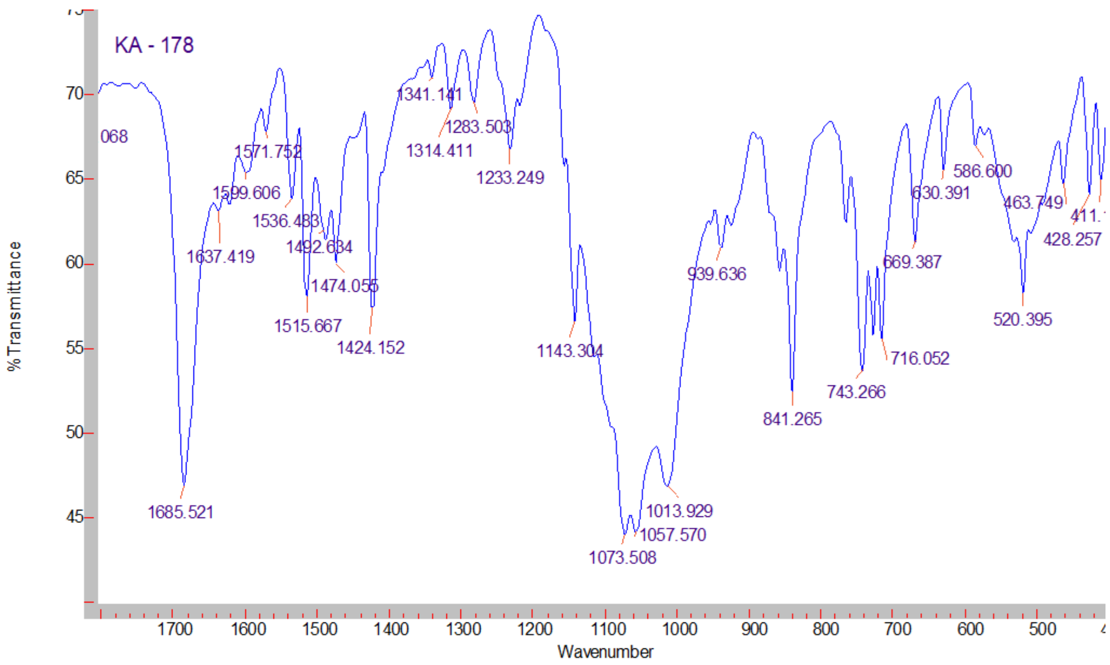

FigureS1a. IR spectrum of complex **7**

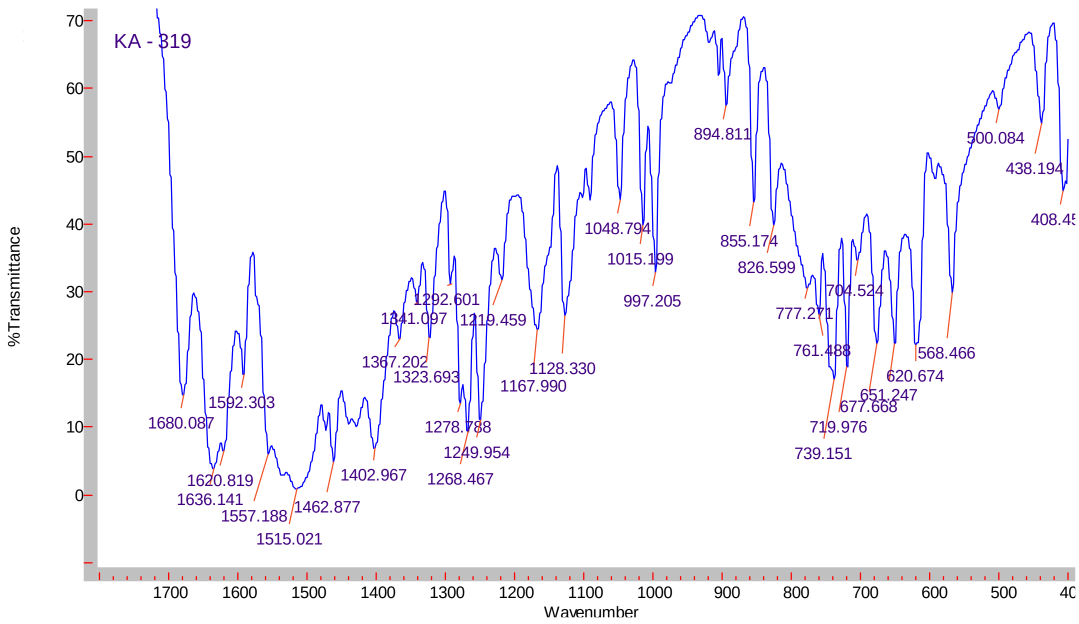

FigureS1b. IR spectrum of compound **3a.**

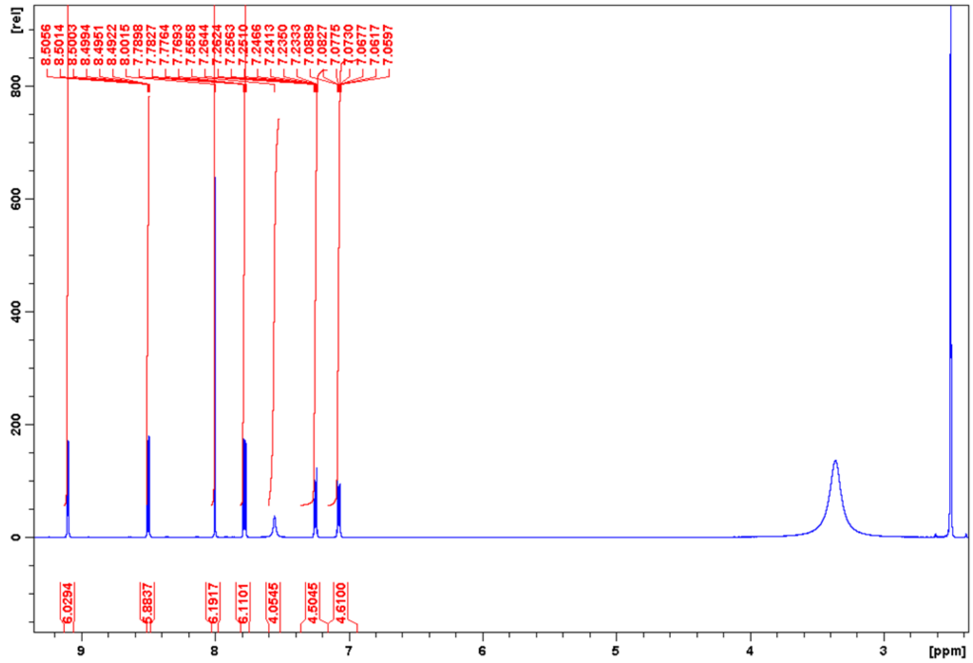

FigureS2. ^{1}H NMR spectrum of complex **5.**

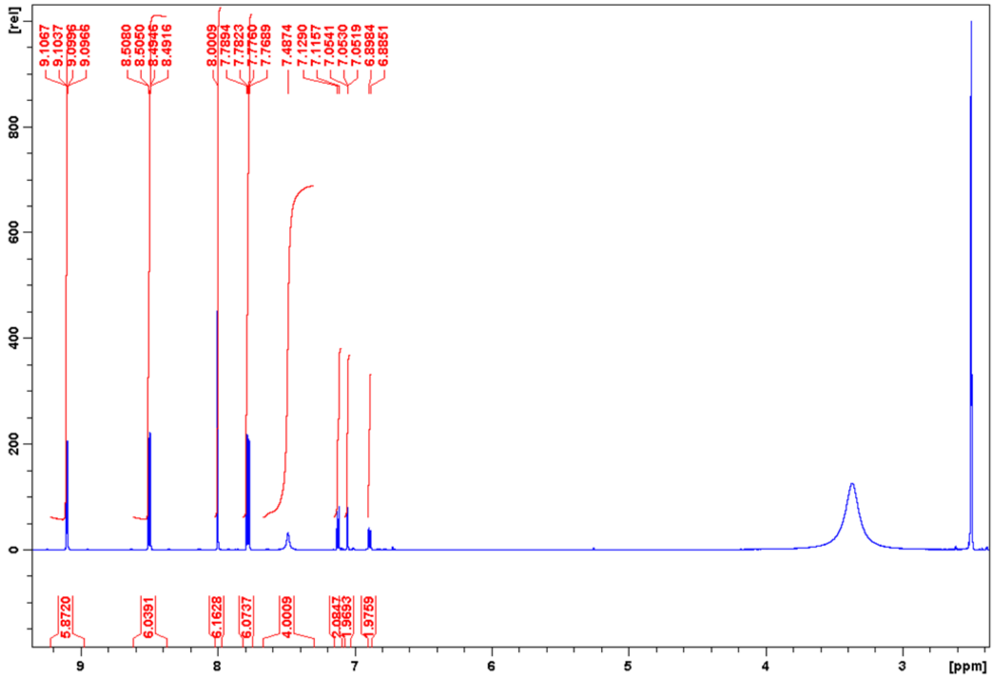

FigureS3. ^{1}H NMR spectrum of complex **6.**

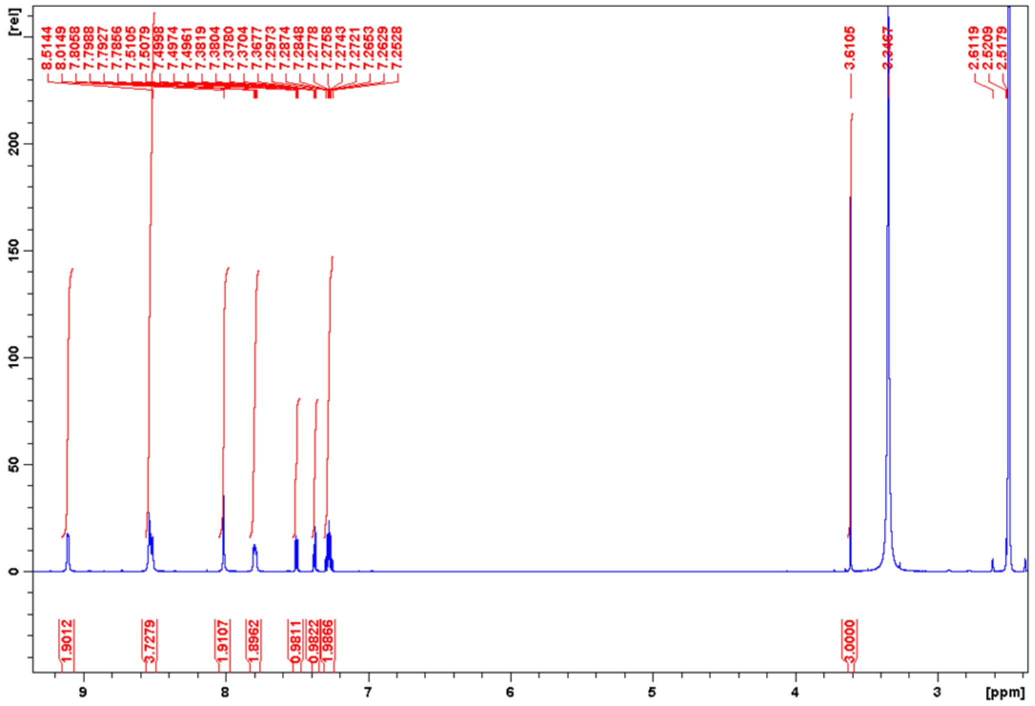

FigureS4. ^{1}H NMR spectrum of complex 7.

Table S1. The most important crystallographic parameters from the crystal structure refinement of complex **7**.

Compound	7
Empirical formula	$C_{20}H_{18}BF_4N_5$
Formula weight	415.20
Temperature	290 K
Crystal system	Monoclinic
Space group	$P2_1/n$
a/Å	7.2843(4)
b/Å	18.5299(10)
c/Å	14.4380(8)
α/°	90
β/°	97.336(6)
γ/°	90
Volume/Å^3	1932.85(19)
Z	4
ρ_{calc}g/cm^3	1.427
μ/mm^{-1}	0.114
F(000)	856.0
Crystal size/mm^3	0.3×0.2×0.15
Radiation	Mo*K*α (λ = 0.71073)
2Θ range for data collection/°	5.69 to 57.982
Reflections collected/ independent	13263/4443
R_{int}/R_{sigma}	0.0397/0.0585
Data/restraints/parameters	4443/0/284
Goodness-of-fit on F^2	1.049
Final *R* indexes [I>=2σ (I)]	R_1 = 0.0653, wR_2 = 0.1445
Final *R* indexes [all data]	R_1 = 0.1332, wR_2 = 0.1802
Largest diff. peak/hole / e Å^{-3}	0.21/-0.21
CCDC	

Table S2. Bond lengths for the crystal structure of **7**

Atom	Atom	Bond Length/Å	Atom	Atom	Bond Length/Å
N8	C9	1.348(3)	N11	C10	1.359(3)
N8	C7	1.393(3)	N11	C12	1.326(4)
N8	C8	1.451(3)	N22	C23	1.360(3)
N1	C2	1.388(3)	N22	C21	1.317(3)
N1	C9	1.332(3)	C10	C23	1.443(4)
F26	B25	1.384(4)	C10	C15	1.410(4)
C2	C7	1.390(4)	C23	C18	1.412(4)
C2	C3	1.385(4)	C15	C16	1.424(4)
F27	B25	1.355(4)	C15	C14	1.395(4)
C9	N9	1.322(3)	C18	C17	1.430(4)
C7	C6	1.379(4)	C18	C19	1.407(4)
C4	C3	1.399(5)	C17	C16	1.339(5)
C4	C5	1.377(5)	C12	C13	1.395(4)
C6	C5	1.378(5)	C20	C19	1.352(5)
F28	B25	1.358(4)	C20	C21	1.389(4)
B25	F29	1.336(5)	C14	C13	1.350(5)

Table S3. Bond Angles for the crystal structure of **7**

Atom	Atom	Atom	Angle/°	Atom	Atom	Atom	Angle/°
C9	N8	C7	107.9(2)	C12	N11	C10	117.6(3)
C9	N8	C8	125.4(2)	C21	N22	C23	118.0(2)
C7	N8	C8	126.5(2)	N11	C10	C23	118.9(2)
C9	N1	C2	108.5(2)	N11	C10	C15	122.0(3)
N1	C2	C7	106.9(2)	C15	C10	C23	119.1(2)
C3	C2	N1	131.6(3)	N22	C23	C10	119.5(2)
C3	C2	C7	121.4(3)	N22	C23	C18	121.4(3)
N1	C9	N8	110.0(2)	C18	C23	C10	119.1(2)
N9	C9	N8	125.9(3)	C10	C15	C16	119.9(3)
N9	C9	N1	124.1(3)	C14	C15	C10	117.8(3)
C2	C7	N8	106.7(2)	C14	C15	C16	122.3(3)
C6	C7	N8	130.9(3)	C23	C18	C17	119.7(3)
C6	C7	C2	122.3(3)	C19	C18	C23	117.7(3)
C5	C4	C3	122.3(3)	C19	C18	C17	122.6(3)
C2	C3	C4	115.7(3)	C16	C17	C18	121.0(3)
C5	C6	C7	116.5(3)	N11	C12	C13	123.7(3)
F27	B25	F26	109.6(3)	C17	C16	C15	121.2(3)
F27	B25	F28	110.0(3)	C19	C20	C21	118.3(3)
F28	B25	F26	108.9(3)	C20	C19	C18	120.1(3)
F29	B25	F26	110.8(3)	C13	C14	C15	120.0(3)
F29	B25	F27	109.6(3)	C14	C13	C12	118.8(3)
F29	B25	F28	107.9(3)	N22	C21	C20	124.5(3)
C4	C5	C6	121.7(3)				
